# Sensory Deprivation during Early Postnatal Period Alters the Density of Interneurons in the Mouse Prefrontal Cortex

**DOI:** 10.1155/2015/753179

**Published:** 2015-06-25

**Authors:** Hiroshi Ueno, Shunsuke Suemitsu, Yosuke Matsumoto, Motoi Okamoto

**Affiliations:** ^1^Department of Medical Technology, Graduate School of Health Sciences, Okayama University, Okayama 700-8558, Japan; ^2^Department of Medical Technology, Kawasaki College of Allied Health Professions, Okayama 701-0194, Japan; ^3^Department of Psychiatry, Kawasaki Medical University, Kurashiki 701-0192, Japan; ^4^Department of Neuropsychiatry, Graduate School of Medicine, Dentistry and Pharmaceutical Sciences, Okayama University, Okayama 700-8558, Japan

## Abstract

Early loss of one sensory system can cause improved function of other sensory systems. However, both the time course and neuronal mechanism of cross-modal plasticity remain elusive. Recent study using functional MRI in humans suggests a role of the prefrontal cortex (PFC) in cross-modal plasticity. Since this phenomenon is assumed to be associated with altered GABAergic inhibition in the PFC, we have tested the hypothesis that early postnatal sensory deprivation causes the changes of inhibitory neuronal circuit in different regions of the PFC of the mice. We determined the effects of sensory deprivation from birth to postnatal day 28 (P28) or P58 on the density of parvalbumin (PV), calbindin (CB), and calretinin (CR) neurons in the prelimbic, infralimbic, and dorsal anterior cingulate cortices. The density of PV and CB neurons was significantly increased in layer 5/6 (L5/6). Moreover, the density of CR neurons was higher in L2/3 in sensory deprived mice compared to intact mice. These changes were more prominent at P56 than at P28. These results suggest that long-term sensory deprivation causes the changes of intracortical inhibitory networks in the PFC and the changes of inhibitory networks in the PFC may contribute to cross-modal plasticity.

## 1. Introduction

The brain can adapt to sensory loss by neuronal plasticity. Blind individuals compensate their lack of visual input by improving sensitivity to auditory or somatosensory inputs. This type of plasticity is known as cross-modal plasticity [1, 2]. Although dynamic cortical reorganization among different sensory areas seemed to be critical for cross-modal plasticity, the molecular and cellular mechanisms underlying it are still poorly understood [1, 2]. The elucidation of the mechanisms underlying cross-modal plasticity provides the clues for developing therapeutic approaches to help sensory recovery and substitution after brain infraction and trauma.

The improved functions of intact sensory systems have been explained by anatomical and functional reorganization of neural circuit in the sensory deprived primary sensory cortex [1, 3–8]. Reorganization of neural circuit in the sensory deprived primary cortex involves formation of aberrant corticocortical or thalamocortical inputs from intact sensory systems, or unmasking of preexisting latent corticocortical and/or thalamocortical inputs. In mice enuleated bilateral eyes immediately after birth, somatosensory axons invade also into the dorsal lateral geniculate nucleus in addition to the ventral lateral nucleus, but the barrel cortex and auditory cortex appear to expand [9]. It indicates cross-modal interactions between somatosensory/auditory and visual area [10–12]. Also in cats, early postnatal visual deprivation induces both somatosensory and auditory cross-modal innervations into the primary visual cortex [4, 13, 14]. Single-unit recordings from a multisensory area called the anterior ectosylvian cortex show that, in visually deprived cats, cortical areas that respond to auditory stimuli expand significantly and neurons in this area are more sharply tuned to auditory spatial localization [13, 15]. Multisensory integration enhances overall perceptual accuracy and saliency [16]. Therefore, it is reasonable to assume that anatomical and functional changes of neuronal networks including the multisensory cortices contribute to improved function of intact sensory systems following loss of one system.

Neuronal plasticity is regulated by higher cognitive functions such as mental arousal and attention [17, 18]. Cross-modal reorganization of different sensory systems may require a coordinated shift of attention from the deprived to the intact sense [19, 20]. A recent study using functional MRI in humans suggests the importance of the prefrontal cortex (PFC) for cross-modal plasticity [21]. The middle temporal complex (MT/MST) specialized for motion perception. In congenitally blind individuals, functional connectivity between MT/MST and dorsal lateral PFC is enhanced, but functional connectivity between MT/MST and primary auditory cortex is not enhanced. The results suggest that cross-modal plasticity may be mediated by the PFC and critical period for cross-modal plasticity may be critical period for plasticity in the PFC but not in the auditory cortex [22].

Converging evidence indicates importance of inhibitory interneurons, in activity-dependent synaptic plasticity and in cross-modal plasticity [9]. Recent studies using experimental models of cross-modal plasticity suggest that changes in the density, laminar distribution, and morphology of interneurons could be a key component in cross-modal plasticity processes following sensory deprivation early in life [9, 23]. The majority of inhibitory interneurons express at least one of the calcium-binding proteins parvalbumin (PV), calbindin (CB), and calretinin (CR), and these proteins have been shown to be excellent markers of distinct interneuron subtypes [24–27]. Together, they account for more than 80% of the total GABAergic interneurons in the rodent PFC [28], highlighting the importance of specific calcium dynamics in interneuronal function. Changes in the expression of calcium-binding proteins can define the functional characteristics of interneurons that impact the inhibitory control of prefrontal output.

Development of GABAergic circuits is a prolonged process that is complete by the end of adolescence [29–32]. The prolonged development of interneurons may constitute a sensitive period where environmental changes can lead to permanent alterations in the inhibitory circuitry. Within the sensory system, neocortical inhibitory networks exhibit experience-dependent maturation [33–38]. However, the influence of experience-dependent plasticity of inhibitory networks and its underlying mechanisms in the PFC is unclear. Destruction of olfactory epithelium by formaldehyde in FVB mice at postnatal day 12 (P12) increases number and neurites complexity of somatostatin neurons in L4 of the barrel cortex and improves tactile sensation [23]. Enucleation of bilateral eyes in hamsters at birth increases PV neurons in L4 and decreases PV neurons and CB neurons in L5 of the primary visual cortex [9]. As a consequence, laminar distribution of PV and CB neurons in the primary visual cortex becomes similar to that in the auditory cortex. Interneurons, particularly CB neurons and PV neurons, have a protracted development reaching their neurochemical and morphological maturity only by the end of adolescence making them very sensitive to sensory experiences [27, 39, 40]. In rodent PFC, the functional maturation of GABAergic inhibition experiences profound changes during adolescence [29–32]. However, it is not clear whether the change of sensory deprivation on interneuron density during postnatal period can be induced in the developing mouse PFC.

In the present study, we examined the effects of sensory deprivation by whisker trimming or dark rearing on the density of PV, CB, and CR neurons in the PFC. We found that sensory deprivation from P0 to P56 increased the density of PV, CB, and CR neurons in the PFC. The results suggest that the changes of interneuron density in the PFC may contribute to cross-modal plasticity.

## 2. Materials and Methods

### 2.1. Animals and Sensory Deprivation

New born C57BL/6N mice were used. All efforts were made to minimize animal's pain and stress. Mice were divided into four groups: (i) whisker trimmed, (ii) dark reared, (iii) dark rearing of whisker trimmed, and (iv) non-treated mice. To examine the effect of somatic sensory deprivation, all principal whiskers on bilateral snouts were trimmed to less than one mm in length everyday using surgical scissors from P2 to P28 or P56. Because whiskers quickly regrow, whisker trimming was performed every day. Due to the short manipulation time, no anesthesia was used. Control mice were also handled every day in the same way but did not have their whiskers trimmed. To examine the effect of visual deprivation, animals were reared in dark condition instead of 12 h light/dark conditions before birth until perfusion at P28 or P56.

The study was carried out in accordance with the National Institute of Health (NIH) Guide for the Care and Use of Laboratory Animals (NIH Publications number 80-23, revised in 1996) and approved by the Committee for Animal Experiments at Okayama University Advanced Research Center. All efforts were made to minimize the number of animals used. Animals were purchased from Charles River Laboratories (Kanagawa, Japan). The animals were housed in cages (3–5 animals per cage) with food and water provided ad libitum under 12 h light/dark conditions at 23–26°C.

### 2.2. Tissue Preparation

The animals were anesthetized with a lethal dose of sodium pentobarbital (Nembutal, Dainippon Sumitomo Pharma, Osaka, Japan; P28 and P56 animals, 100 mg/kg, i.p.) and transcardially perfused with ice-cold 0.01 M phosphate buffer (PB) for 2 min, followed by 4% paraformaldehyde and 0.2% picric acid in 0.1 M PB, pH 7.4, for 10 min. The brains were dissected and postfixed overnight in the same fixative at 4°C. They were then cryoprotected by incubation in 15% sucrose for 7 h followed by 30% sucrose for 20 h at 4°C. The brains were then frozen in O.C.T. compound (Tissue-Tek, Sakura Finetek, Tokyo, Japan) by freezing in dry ice-cold normal hexane. Serial 40 *μ*m coronal sections were prepared on a cryostat (CM-1900, Leica, Wetzlar, Germany) at −20°C. Every section at the level of the PFC was collected and placed in ice-cold phosphate-buffered saline (PBS).

### 2.3. Immunohistochemistry

The cryostat sections were washed in PBS, incubated in 3% H_2_O_2_ in PBS for 15 min, washed again in PBS, and treated with 0.1% TritonX-100 in PBS at room temperature for 15 min. After three washes in PBS, the sections were incubated with 10% normal goat serum (Funakoshi Corporation, Tokyo, Japan) in PBS at room temperature for 1 h. After three washes in PBS, the sections were incubated with primary antibody mouse anti-parvalbumin (clone PARV-19, P3088, Sigma-Aldrich Japan, Tokyo, Japan, 1 : 20,000), mouse anti-calbindin D28k (clone CB-955, C9848, Sigma-Aldrich Japan, Tokyo, Japan, 1 : 2,000), rabbit anti-calretinin (AB5054, Millipore, Tokyo, Japan, 1 : 2,000), and mouse anti-NeuN (clone A60, MAB377, Millipore, Tokyo, Japan, 1 : 2,000) in PBS overnight at 4°C. After being washed in PBS, the sections were incubated with secondary antibody biotin-conjugated rabbit anti-mouse IgG (MA01742-3049, Fitzgerald Industries International, Concord, MA, USA, 1 : 1,000) and biotin-conjugated goat anti-rabbit IgG (BA-1000, Vector Laboratories, CA, USA, 1 : 1,000). After three washes in PBS, the sections were incubated with streptavidin-biotin-peroxidase conjugate (VECSTAIN ABC kit, Vector Laboratories, Funakoshi Co., Tokyo, Japan) for 1 h at room temperature. Immunohistochemistry was visualized by incubating the samples in PBS supplemented with 0.03% 2,3-diaminobenzidine tetrahydrochloride (DAB, Sigma, St. Louis, MO, USA), 0.01% H_2_O_2_, and 0.3% ammonium nickel sulfate hexahydrate. Sections were washed in PBS, mounted onto glass slides, dehydrated, cleaned with xylene, and coverslipped.

### 2.4. Quantification of Interneurons

For preparation of digital images, light microscopic images were captured by LuminaVision software (version 2.4.0, Mitani Corporation, Fukui, Japan), and brightness and contrast were slightly adjusted.

**Figure 1:**
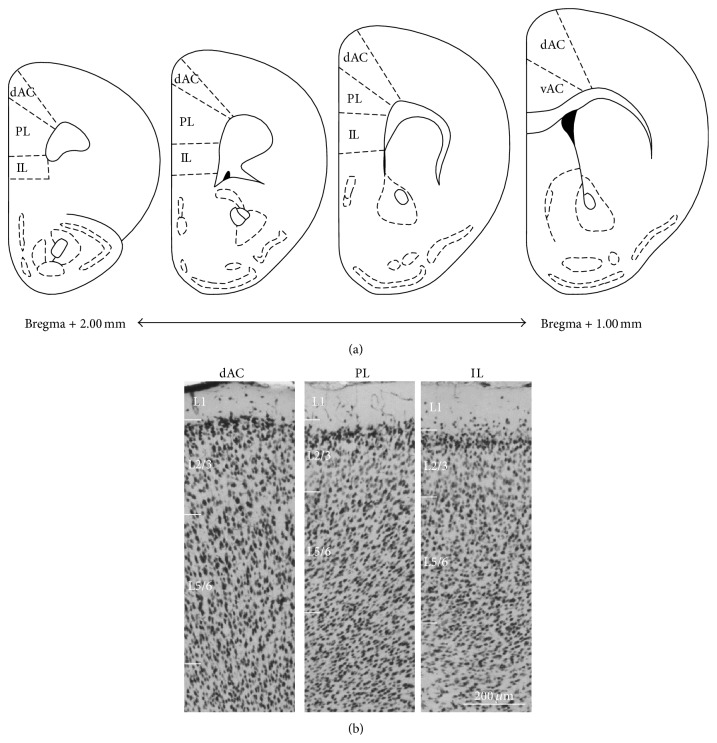
Anatomical characteristics of the mouse PFC. (a) Diagram depicting parcellated subareas of the PFC (PL, IL, dAC, and vAC). (b) Light micrograph illustrating the laminar distribution of NeuN-positive neurons of the PFC. Cytoarchitectural characteristics of the dAC, PL, and IL region. Scale bars = 200 *μ*m.

The medial regions of PFC (prelimbic cortex (PL), infralimbic cortex (IL), and dorsal regions of anterior cingulate cortex (dAC)) were parcellated according to cytoarchitectonic criteria [41], with reference to [42]. All cytoarchitectural boundaries were assessed at 4x magnification (Figure 1). To visualize pyramidal neuron somata, we used NeuN immunoreactivity. NeuN is thought to be a pan-neuronal marker. The PL, IL, and dAC regions were identified using NeuN staining. The border between the PL and dAC is marked by a widening of layer 5 (L5) and an increase in the density of L3 cells in the dAC as compared to the PL. The posterior border of the dAC and ventral regions of anterior cingulate cortex (vAC) is defined by an increase in the density of cells in, L2 and by the presence of clearly distinguishable L3. The border between IL and PL is made principally on the basis of the transition between L1–L3; the most superficial cells in L2 of IL extend into L1 whereas the boundary between L1 and L2 in the PL is much more distinct. L2 cells are more densely packed in the PL as compared to IL. L2/3 and L5/6 were measured separately (mouse PFC lacks L4). Beginning with the first section containing white matter, prefrontal cortical areas were parcellated on each section collected until the first section in which the genu of the corpus callosum appeared. This results in parcellation of both hemispheres in 25 sections per animal. Five sections (1 in 5 series) through the entire region were selected and stained with NeuN, and volume was calculated. The average number of immunoreactive neurons at each level in a region was obtained from bilateral counts in a single section and estimated number of neurons calculated according to Abercrombie's formula [43]. For quantification of interneurons and measurement of each region, immunohistochemical images captured by LuminaVision software were analyzed using NIH imageJ software (NIH, Bethesda, MD; http://rsb.info.nih.gov/nih-image/). Estimations of neural density (cells/mm^2^) were carried out.

### 2.5. Data Analysis

The quantification was performed by an observer who was blinded to the sensory deprivation. Data are expressed as mean ± S.E.M. of six animals per group. Statistical comparisons were performed using ANOVA or Mann-Whitney *U* test, and statistical significance was set at <0.05.

## 3. Results

**Figure 2:**
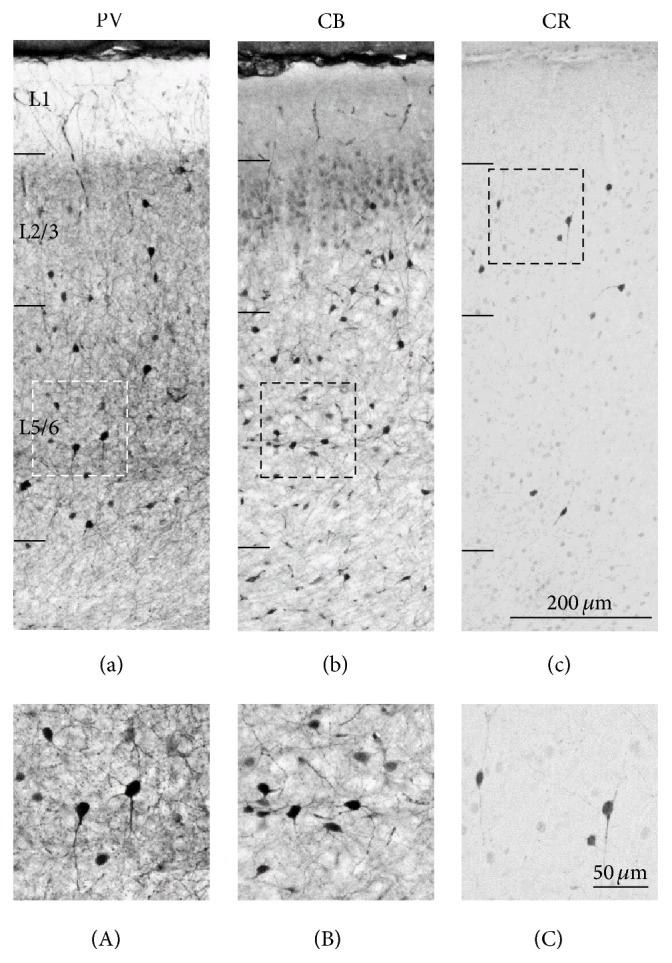
Localization of interneuron in the mouse PFC. Representative light micrograph showing laminar distribution of PV (a), CB (b), and CR (c) neurons in the PL. (a) PV neurons are located in L2/3 and L5/6. (b) CB neurons are mainly located in L2/3 and L5/6. The principal neurons in L2 are weakly immunopositive to CB. (c) CR neurons are mainly located in L2/3. (A)–(C) Higher magnification images of interneurons in the PL. Scale bars = 200 *μ*m in (c) (applies to (a)–(c)); 50 *μ*m in (C) (applies to (A)–(C)).

As shown in Figure 2, all interneuron subtypes were present in the mouse PFC at P28. In the PL, PV neurons were located in both L2/3 and L5/6 (Figure 2(a)). The processes of PV neurons were distributed through all layers. Also CB neurons were present in both L2/3 and 5/6 (Figure 2(b)). Although the principal neurons in L2 are weakly immunopositive to CB [44], CB positive interneurons were darkly stained compared to principal neurons. The majority of CB neurons were multipolar, but bipolar neurons were also observed (Figure 2(B)). CR neurons were more abundant in L2/3 than L5/6 (Figure 2(c)). They displayed bipolar arbor-like morphology (Figure 2(C)). These distribution patterns of calcium-binding proteins were consistent with previous literature [28, 44–47]. In order to evaluate whether specific interneuronal population might be more susceptible to sensory deprivation, we analyzed the density of PV, CB, and CR neurons.

### 3.1. Changes of the Density of PV Neurons in the Primary Sensory Cortices at P28

**Figure 3:**
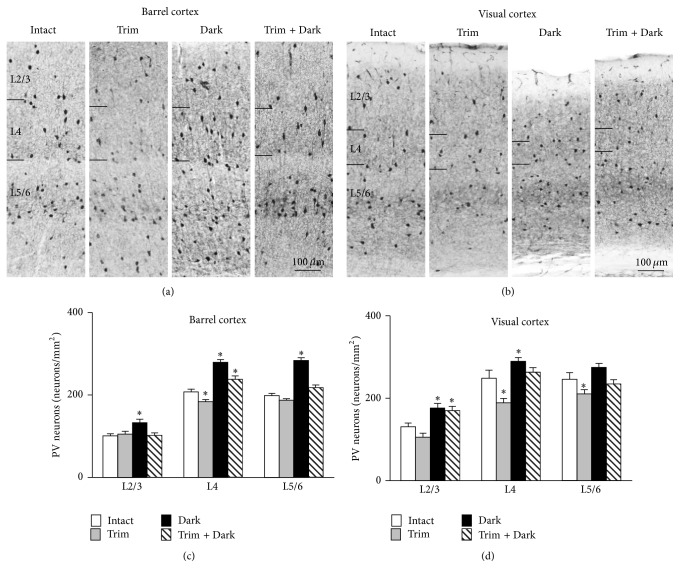
Effects of sensory deprivation on the appearance of PV neurons in the barrel cortex and primary visual cortex. Coronal sections of the barrel cortex (a) and visual cortex (b) stained with anti-PV antibody at P28 are shown. Upper side is the pial surface. Intact: intact control, trim: whisker trimming, dark: dark rearing, and trim + dark: whisker trimming plus dark rearing. Scale bars = 100 *μ*m (applies to (a), (b)). Changes of the density of PV neurons in each layer of the barrel cortex (c) and visual cortex (d) at P28. Whisker trimming decreased PV neurons in L4 of the barrel cortex. Dark rearing increased PV neurons in L2/3 and L4 of visual cortex. Data are expressed as mean density S.E.M. (*n* = 6 per group). Abbreviations are the same as in (a) and (b). ^*∗*^ *P* < 0.05 compared with intact mice.

Bilateral trimming of all principal whiskers significantly decreased the density of PV neurons in L4 of the barrel cortex at P28 (Figures 3(a) and 3(c)). The decrease of PV neuron density in L4 by whisker trimming in neonatal mice was consistent with the previous study [48]. Whisker trimming decreased PV neuron density in L4 and 5/6 of the primary visual cortex (Figure 3(d)).

At P28 dark rearing increased the density of PV neurons in L2/3 and 4 in the primary visual cortex (Figures 3(b) and 3(d)). In addition, dark rearing caused atrophy of the primary visual cortex as described in hamsters enucleated bilateral eyes at birth [9]. Dark rearing increased PV neuron density in all layers of the barrel cortex (Figures 3(a) and 3(c)). A possible reason of the increase of PV neuron density in the barrel cortex is increased whisking activity following dark rearing. To examine this, we performed dark rearing in whisker trimmed mice. Whisker trimming cancelled the increase of PV neuron density in L2/3 and 5/6 of the barrel cortex. However, the increase of PV neuron density in L4 was still significant in whisker trimmed mice (Figures 3(a) and 3(c)). Whisker trimming also cancelled the increase of PV neuron density in L4 of the primary visual cortex. However, the increase of PV neuron density in L2/3 was not cancelled (Figure 3(d)). Furthermore, the atrophy of the primary visual cortex by visual deprivation was not rescued by whisker trimming (Figures 3(b) and 3(d)).

### 3.2. Effects of Sensory Deprivation on the Density of PV Neurons in the PFC at P28

**Figure 4:**
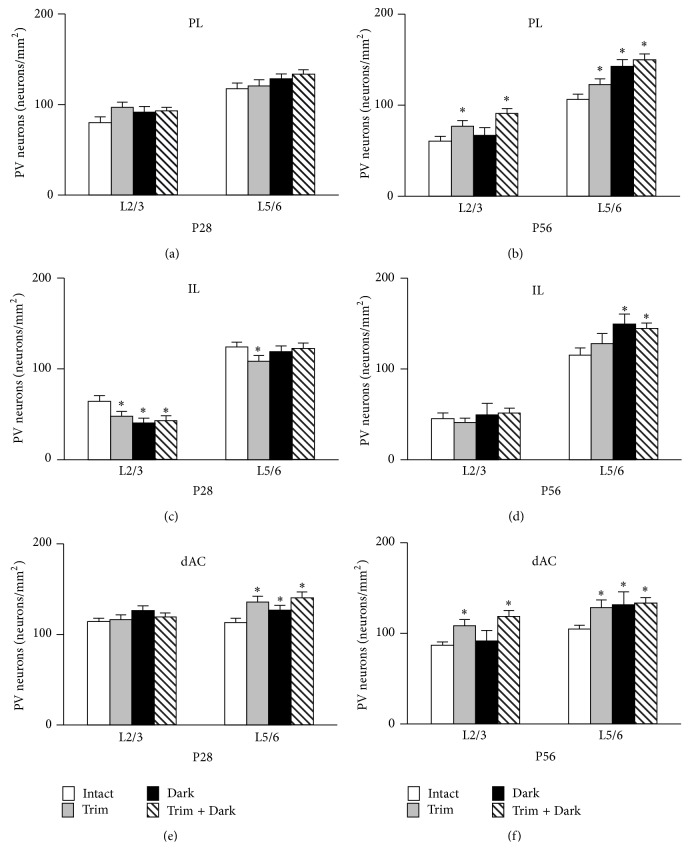
Sensory deprivation changes PV neuron density in the PFC. The density of PV neurons in the PL (a, b), IL (c, d), and dAC (e, f). PV neuron density at P28 (a, c, e) and P58 (b, d, f) in each area are shown. (a)-(b) The density of PV neurons was increased in L5/6 by sensory deprivation at P56. (c)-(d) The density of PV neurons in L2/3 was decreased at P28 by sensory deprivation, but it was increased in L5/6 at P56. (e)-(f) The density of PV neurons in L5/6 was increased with sensory deprivation at both P28 and P56. Data are expressed as mean density S.E.M. (*n* = 6 per group). Abbreviations are the same as in Figure 4. ^*∗*^ *P* < 0.05 compared with intact mice.

There was no change in the density of PV neurons between intact mice and sensory deprived mice in the PL (Figure 4(a)). In L2/3 of the IL, whisker trimming, dark rearing, or dark rearing of whisker trimmed mice significantly decreased PV neuron density (Figure 4(c)). Moreover, whisker trimming significantly decreased PV neuron density in L5/6 of the IL (Figure 4(c)). Whisker trimming, dark rearing, or dark rearing of whisker trimmed mice significantly increased PV neuron density in L5/6 but not in L2/3 of the dAC (Figure 4(e)).

### 3.3. Effects of Sensory Deprivation on the Density of PV Neurons in the PFC at P56

**Figure 5:**
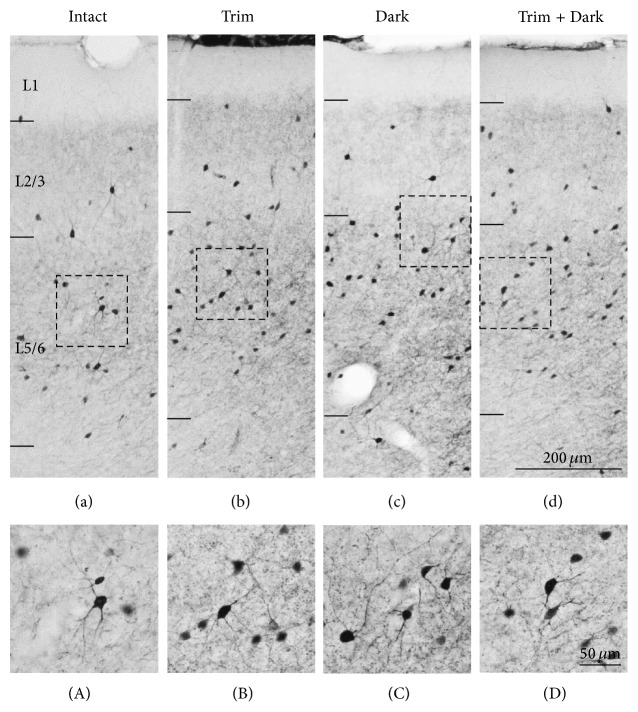
Effects of sensory deprivation on PV neurons in the PL at P56. Whisker trimmed mice (b), dark reared mice (c), and whisker trimmed and dark reared mice (d) have more PV neurons than intact mice (a). (A)–(D) Higher magnification images of PV neurons in the PL. Intact: intact control, trim: whisker trimming, dark: dark rearing, and trim + dark: whisker trimming plus dark rearing. Scale bars = 200 *μ*m in (d) (applies to (a)–(d)); 50 *μ*m in (D) (applies to (A)–(D)).

Representative examples of PV staining of the PL from each group are shown in Figure 5. The effect of sensory deprivation from P0 to P56 in the PL was different compared with P28. Whisker trimming or dark rearing of whisker trimmed mice significantly increased PV neuron density both in L2/3 and 5/6 of the PL (Figure 4(b)). Dark rearing increased PV neuron density in L5/6 of the PL (Figure 4(b)). Dark rearing or dark rearing of whisker trimmed mice increased PV neuron density in L5/6 of the IL (Figure 4(d)). Whisker trimming or dark rearing of whisker trimmed mice significantly increased PV neuron density in both L2/3 and L5/6 of the dAC (Figure 4(f)). Dark rearing increased PV neuron density in only L5/6 of the dAC (Figure 4(f)).

### 3.4. Effects of Sensory Deprivation on the Density of CB Neurons in the PFC at P28

**Figure 6:**
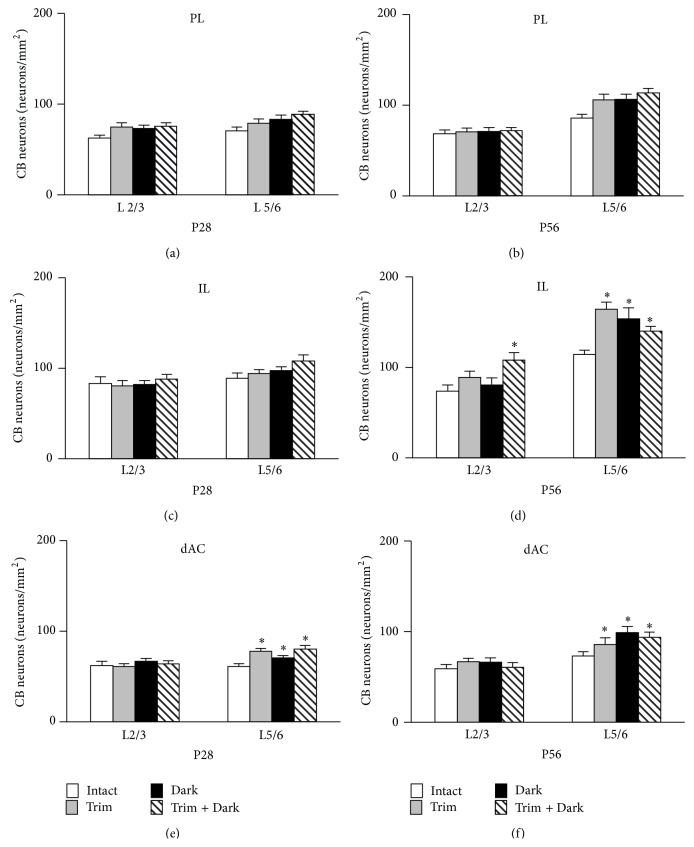
Sensory deprivation changes CB neuron density in the PFC. The density of CB neurons in the PL (a, b), IL (c, d), and dAC (e, f). CB neuron density at P28 (a, c, e) and P58 (b, d, f) in each area are shown. (a)-(b) The density of CB neurons was not affected in sensory deprived mice in the PL. (c)-(d) At P56 the density of CB neurons in L5/6 was increased by sensory deprivation. (e)-(f) The density of CB neurons in L5/6 was increased with sensory deprivation at P28 and P56. Data are expressed as mean density S.E.M. (*n* = 6 per group). Abbreviations are the same as in Figure 4. ^*∗*^ *P* < 0.05 compared with intact mice.

We found no difference in CB neuron density both in the PL and in the IL for mice sensory deprived compared with intact mice (Figures 6(a) and 6(c)). In contrast to the PL and IL, we found a significant increase in the density of CB neurons in the dAC L5/6 of sensory deprived mice (Figure 6(e)).

### 3.5. Effects of Sensory Deprivation on the Density of CB Neurons in the PFC at P56

Similar to the finding at P28 no significant difference in the density of CB neurons was found between intact mice and sensory deprived mice in the PL at P56 (Figure 6(b)). Whisker trimming or dark rearing significantly increased CB neuron density in L5/6 of the IL (Figure 6(d)). Dark rearing of whisker trimmed mice significantly increased CB neuron density in both L2/3 and 5/6 of the IL (Figure 6(d)). Similar to finding at P28, whisker trimming, dark rearing, or dark rearing of whisker trimmed mice significantly increased CB neuron density in L5/6 of the dAC (Figure 6(f)).

### 3.6. Effects of Sensory Deprivation on the Density of CR Neurons in the PFC at P28

**Figure 7:**
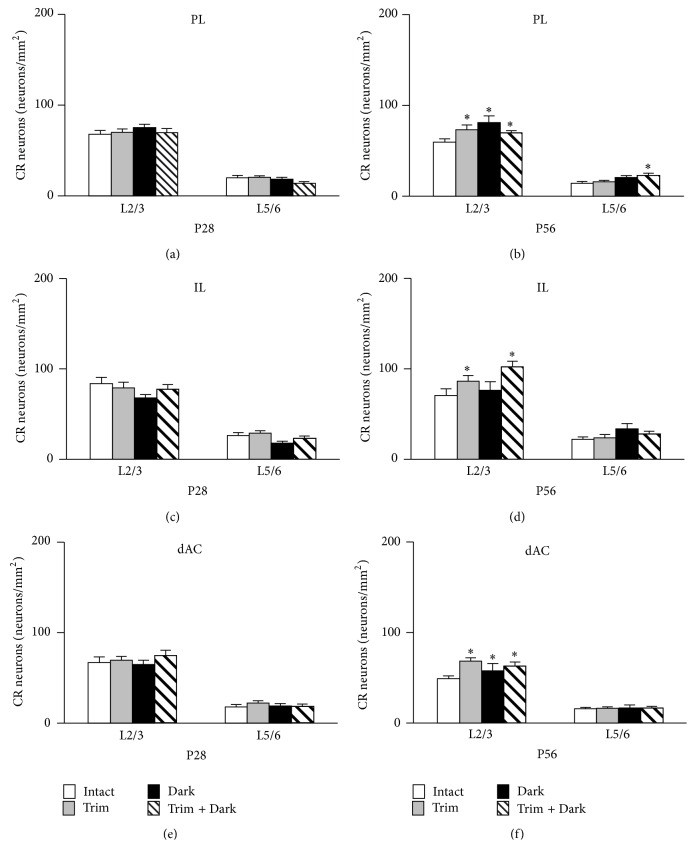
Sensory deprivation changes CR neuron density in the PFC. The density of CR neurons in the PL (a, b), IL (c, d), and dAC (e, f). CR neuron density at P28 (a, c, e) and P58 (b, d, f) in each area are shown. (a)-(b) There was no effect of sensory deprivation on the density of CR neurons in the PFC at P28. The density of CR neurons in L2/3 was increased by sensory deprivation at P56. (c)-(d) At P56 the density of CR neurons in L2/3 was increased by whisker trimming and dark rearing. (e)-(f) The density of CR neurons in L2/3 was increased by sensory deprivation at P56. Data are expressed as mean density S.E.M. (*n* = 6 per group). Abbreviations are the same as in Figure 4. ^*∗*^ *P* < 0.05 compared with intact mice.

There was no significant difference in CR neuron density between sensory deprived mice and intact mice in the PFC at P28 (Figures 7(a), 7(c), and 7(e)).

### 3.7. Effects of Sensory Deprivation on the Density of CR Neurons in the PFC at P56

Whisker trimming, dark rearing, or dark rearing of whisker trimmed mice significantly increased CR neuron density in L2/3 of the PL (Figure 7(b)). Dark rearing of whisker trimmed mice also increased CR neuron density in L5/6 of the PL (Figure 7(b)). Whisker trimming or dark rearing of whisker trimmed mice increased CR neuron density in L2/3 of the IL (Figure 7(d)). In contrast, the upregulation of the density of CR neurons by only dark rearing was not observed in the IL (Figure 7(d)). Whisker trimming, dark rearing, or dark rearing of whisker trimmed mice significantly increased CR neuron density in L2/3 but not in L5/6 of the dAC (Figure 7(f)).

## 4. Discussion

### 4.1. Changes of the Interneuron Density in the Primary Sensory Cortices by Sensory Deprivation

PV neurons appear to be coincident with the onset of critical period of the formation of receptive field in the barrel cortex [49] and for ocular dominance plasticity in the primary visual cortex [35, 50, 51]. Sensory deprivation retards the appearance of PV neurons and maturation of synapse formation by PV neurons in the barrel cortex [48] and the primary visual cortex [33, 52]. PV neurons are more sensitive to sensory deprivation than excitatory neurons [53]. Therefore, we at first examined the appearance of PV neurons in the barrel cortex and primary visual cortex to confirm that whisker trimming or/and dark rearing induces cross-modal plasticity in the primary sensory cortices.

Dark rearing increased PV neurons in the barrel cortex (Figure 3(c)). This is likely in most part due to increase of whisking activity because the increase of PV neuron density in L2/3 and L5/6 was cancelled by whisker trimming. However, the increase of PV neuron density in L4 was not cancelled by whisker trimming (Figure 3(c)). The latter suggests the presence of corticocortical or corticothalamocortical interactions between the barrel cortex and other sensory cortices (e.g., primary auditory cortex) or multimodal association cortices. Dark rearing also increased PV neuron density may be in part due to atrophy of the primary visual cortex (Figure 3(d)). However, whisker trimming cancelled the increase of PV neuron density in L4 without protecting the atrophy of the primary visual cortex (Figures 3(b) and 3(d)). The result suggests that the increase of PV neurons in L4 is in part caused by recruitment or unmasking of somatosensory inputs to the primary visual cortex. However, the increase of PV neurons in L2/3 is unlikely due to unmasking or recruitment of somatosensory input because it was not cancelled by whisker trimming.

Whisker trimming decreased PV neurons in L4 and L5/6 in the primary visual cortex (Figure 3(d)). The decrease of PV neurons by whisker trimming was cancelled by dark rearing (Figure 3(d)). One possible explanation for the decrease of PV neurons by whisker trimming is competitive interaction or reciprocal inhibition between visual and somatosensory thalamocortical projections to the visual cortex. Presence of latent somatosensory input to the visual cortex is shown in adult mice [54]. Somatosensory input to the visual cortex is supposed to dominate visual input in neonatal mice because maturation of receptive field in the barrel cortex completes in the second postnatal week [49] whereas formation of ocular dominance column in the primary visual cortex completes in the fourth postnatal week [35, 50, 51]. Therefore, neonatal visual deprivation may enhance somatosensory input to the visual cortex whereas somatosensory deprivation attenuates it. Consistent with this notion, whisker trimming plus dark rearing increased PV neuron density in L2/3 (Figure 3(d)). It is difficult to explain this by recruitment or unmasking of intact sensory inputs to the primary visual cortex. It suggests the presence of corticocortical or corticothalamocortical interactions between the primary visual cortex and other sensory cortices (e.g., primary auditory cortex) or association cortices.

Together, whisker trimming or/and dark rearing induced unimodal and cross-modal changes of PV neuron density in the primary sensory cortices that were in part considered to be due to corticocortical interactions.

### 4.2. Changes of the Interneuron Density in the PFC by Sensory Deprivation

The expression of calcium-binding proteins PV, CB, and CR was differently regulated in the PFC during developmental period [29–32]. PV expression is very low at early postnatal stages and is significantly increased by early adulthood. In contrast, the expression of CR is reduced from juvenile to early adulthood, whereas the expression of CB remains unaltered during development period [32].

The density of CB neurons significantly increased in L5/6, whereas the density of CR neurons increased in L2/3 of the PFC of the sensory deprived mice at P56. CB neurons innervate distal dendritic shafts and dendritic spines of pyramidal neurons. Recent in vivo studies using two-photon laser scanning microscopy and fluorescently tagged gephyrin have demonstrated that pruning of inhibitory synapse terminals at distal dendritic spines of pyramidal neurons is major mechanisms of ocular dominance plasticity in the adult visual cortex [55, 56]. Although both studies have not identified interneurons providing inhibitory synapse terminals at distal dendrites, the most possible candidates are CB/somatostatin neurons. If this is the case, sensory deprivation may increase inhibitory synapse terminals on distal dendrites of pyramidal neurons in the PFC. CR neurons mainly innervate dendritic shafts of pyramidal neurons. Furthermore, CR neurons innervate other interneurons, particularly CB neurons, in supragranular layer of cerebral cortex, and their excitation causes disinhibition of pyramidal neurons [57–60]. A recent study has demonstrated that inhibitory inputs on dendrites are more powerful than inhibitory inputs to perisomatic regions for inhibiting firing of CA1 pyramidal neurons in the hippocampus [61]. Therefore, the increase of L5/6 CB and L2/3 CR neuron densities by sensory deprivation may reflect enhanced synaptic inhibition on pyramidal neuron dendrites and reduced excitation of pyramidal neurons. Alternatively the increase of CB and CR neurons may reflect compensatory mechanism for increased excitatory inputs to protect excessive excitation of pyramidal neurons. The density of PV neurons increased in L2/3 and L5/6 of the PFC of the sensory deprived mice, compared to intact mice, at P56. The protein level of PV is lowest in juveniles and increases during adolescence to levels similar to those observed in adulthood [32]. The increased PV expression during adolescence is an activity-driven event at a time when metabolic demand of energy increased [62]. An increase of PV neurons in the PFC by sensory deprivation has not been reported. PV basket neurons control synchronous firing and output of pyramidal neurons [63]. Therefore, the results suggest that somatosensory deprivation and visual deprivation enhance the control by PV neurons on synchronous firing of pyramidal neurons in the PFC although electrophysiological analysis is required to clarify these points. And, the alteration of PV neurons by sensory deprivation was likely the result of an increase of the survival rate of PV neurons. Anyway the alterations of the interneuron density of the PFC in sensory deprived mice indicate that alternative pathway for cross-modal plasticity may be present.

### 4.3. Regional Variability in Sensory Deprived Alteration of Interneurons

The change of the density of PV, CB, and CR interneurons after sensory deprivation was similar among the PL, IL, and dAC. These results confirm our prediction that early sensory deprivation interferes with the development of distinct interneuron subtypes in the PL, IL, and dAC.

The PFC receives multimodal corticocortical projections from auditory, somatosensory, visual, gustatory, and limbic cortices and has a role as nodal station of cortical networks [64]. Pyramidal neurons in L5/6 of the PFC project to L2/3 of the primary sensory cortices [57]. Therefore, it seems likely that the increase of PV neuron density around L5/6 pyramidal neurons by sensory deprivation modifies outputs from the PFC to the primary sensory cortices. The increase of PV neurons in L2/3 of the primary visual cortex by somatosensory plus visual deprivation (Figure 3(d)) may be a result of increased input from the prefrontal cortex. On the neuroanatomical level, the dorsal and ventral PFC has different connections with other brain regions. The dAC predominantly has reciprocal connections with motor, somatosensory, visual, and retrosplenial cortices. The PL and IL are distinguishable from dAC by relatively greater connectivity with the hippocampus and other limbic structures [64]. The dAC contributes to the formation of emotional and motivational behaviors, attention, and visceral function by interactions with the PL and IL. Attention is required for certain forms of associative learning [17] and involves the function of the dAC in rodents [65–67].

### 4.4. Possible Role of PFC in Cross-Modal Plasticity

Recent studies have shown that thalamocortical projection is already well-segregated into eye-specific bands before the onset of critical period for ocular dominance plasticity [68, 69]. In the rat barrel cortex, supragranular neurons selectively respond to a single principal whisker before the critical period for the formation of receptive field in L2/3 [70]. Therefore, a role of experience-dependent plasticity may be sharpening receptive fields and balancing excitatory and inhibitory inputs to principal neurons [71]. Another possible role of sensory experience is enhancement of anatomical and functional connections between sensory cortices and association cortices that integrate multisensory inputs [71]. In this context, it is not surprising that the PFC mediates sensory information to association cortices or primary sensory cortices to compensate loss of a sensory input. Our results indicate that sensory deprivation modifies inhibitory control of synaptic inputs to pyramidal neurons and of outputs from pyramidal neurons in the PFC and suggest that the anatomical and functional changes of inhibitory neuronal circuit in the PFC may contribute to cross-modal plasticity.

## 5. Conclusions

We show for the first time somatosensory or/and visual deprivation increased PV, CB, and CR neuron densities in the PFC. The results suggest that sensory deprivation enhances inhibitory control of synaptic inputs to pyramidal neurons and of synchronous firing of pyramidal neurons in the PFC, and anatomical and functional changes of inhibitory neuronal circuit in the PFC may contribute to cross-modal plasticity. And, these findings indicate that sensory input is needed for the normal development of interneurons in the PFC.
